# Rapid systematic review on risks and outcomes of sepsis: the influence of risk factors associated with health inequalities

**DOI:** 10.1186/s12939-024-02114-6

**Published:** 2024-02-21

**Authors:** Siân Bladon, Diane Ashiru-Oredope, Neil Cunningham, Alexander Pate, Glen P Martin, Xiaomin Zhong, Ellie L Gilham, Colin S Brown, Mariyam Mirfenderesky, Victoria Palin, Tjeerd P van Staa

**Affiliations:** 1 Centre for Health Informatics & Health Data Research UK North, Division of Informatics, Imaging and Data Science, School of Health Sciences, Faculty of Biology, Medicine and Health, The University of Manchester, Manchester Academic Health Science Centre, Manchester, M13 9PL UK; 2 Healthcare-Associated Infection (HCAI), Fungal, Antimicrobial Resistance (AMR), UKHSA, London, SW1P 3JR UK; 3 NIHR Health Protection Unit in Healthcare-Associated Infection & Antimicrobial Resistance, Imperial College London, London, UK; 4 Maternal and Fetal Health Research Centre, Division of Developmental Biology and Medicine, The University of Manchester, Manchester, M13 9WL UK; 5 School of Pharmacy, University of Nottingham, Nottingham, NG7 2RD UK

**Keywords:** Sepsis, Antimicrobial resistance, Health inequalities, Socioeconomic status, Deprivation, Ethnicity, Communities, Maternal

## Abstract

**Background and aims:**

Sepsis is a serious and life-threatening condition caused by a dysregulated immune response to an infection. Recent guidance issued in the UK gave recommendations around recognition and antibiotic treatment of sepsis, but did not consider factors relating to health inequalities. The aim of this study was to summarise the literature investigating associations between health inequalities and sepsis.

**Methods:**

Searches were conducted in Embase for peer-reviewed articles published since 2010 that included sepsis in combination with one of the following five areas: socioeconomic status, race/ethnicity, community factors, medical needs and pregnancy/maternity.

**Results:**

Five searches identified 1,402 studies, with 50 unique studies included in the review after screening (13 sociodemographic, 14 race/ethnicity, 3 community, 3 care/medical needs and 20 pregnancy/maternity; 3 papers examined multiple health inequalities). Most of the studies were conducted in the USA (31/50), with only four studies using UK data (all pregnancy related). Socioeconomic factors associated with increased sepsis incidence included lower socioeconomic status, unemployment and lower education level, although findings were not consistent across studies. For ethnicity, mixed results were reported. Living in a medically underserved area or being resident in a nursing home increased risk of sepsis. Mortality rates after sepsis were found to be higher in people living in rural areas or in those discharged to skilled nursing facilities while associations with ethnicity were mixed. Complications during delivery, caesarean-section delivery, increased deprivation and black and other ethnic minority race were associated with post-partum sepsis.

**Conclusion:**

There are clear correlations between sepsis morbidity and mortality and the presence of factors associated with health inequalities. To inform local guidance and drive public health measures, there is a need for studies conducted across more diverse setting and countries.

**Supplementary Information:**

The online version contains supplementary material available at 10.1186/s12939-024-02114-6.

## Introduction

Sepsis is “life-threatening organ dysfunction caused by a dysregulated host immune response to an infection” [1]. A 2015 study estimated the in-hospital mortality rate for sepsis in UK hospitals to be around 30% [2]. As well as high mortality rates, sepsis survivors often experience longer-term mental and physical health problems and are at high risk of post-discharge hospital readmission or death [3–5]. Risk factors for developing sepsis include frailty, immunocompromised status, recent surgical procedures, and comorbidities such as cancer, kidney disease, lung disease and diabetes [6–8]. The risk of contracting sepsis increases with age, with many sepsis cases occurring in people over the age of 65 [9]. Additionally, there is a higher risk of sepsis in neonates and women who are pregnant or have recently given birth.

Bacterial infections are the most common cause of sepsis and therefore antibiotics are widely used for treatment. A 2022 report published by the United Kingdom (UK) Academy of Medical Royal Colleges (AMRC) outlined recommendations for the recognition and early management of sepsis [10]. An aspect the report did not address, however, was the impact of health inequalities on sepsis recognition, management and outcomes. Inequalities can impact life expectancy, access to healthcare and general health status. Factors that are associated with these disparities include level of deprivation, ethnicity and belonging to more vulnerable groups within society, for example people experiencing homelessness [11]. In 2021, the National Healthcare Inequalities Programme was set up and developed the Core20PLUS5 approach, with the aim of supporting the National Health Service and local authorities in reducing health inequalities [11]. Core20 refers to populations living in the most deprived 20% of areas according to the Index of Multiple Deprivation (IMD). PLUS refers to population groups identified at local level that could include ethnic minority groups, coastal communities, populations defined as having a protected characteristic under the Equality Act 2010 or belonging to an inclusion health group, amongst others. ‘5’ refers to five clinical areas of importance, which are maternity, severe mental illness, chronic respiratory disease, early cancer diagnosis, and hypertension case-finding.

Variations in rates of antimicrobial resistant infections and microorganisms (associated with higher mortality rates in sepsis [12]) have been reported in the UK amongst different ethnic groups and levels of deprivation [13]. A recent study reported increased odds of non-COVID 19 related sepsis and increased mortality in more socioeconomically deprived people during the pandemic [14]. In the face of increasing resistance to antimicrobials globally, knowing who is at greatest risk of developing sepsis may not only improve patient outcomes but help target the use of antimicrobials more effectively.

A 2019 systematic review assessed the link between race and socioeconomic status and sepsis outcomes. However, they only included studies conducted in the USA [15]. The purpose of this review, therefore, was to identify studies from all high- income countries that have assessed additional factors associated with health inequality. The aims of this rapid review were (i) to summarise the literature that investigated health inequalities and sepsis incidence and mortality outcome and (ii) to provide an evidence base for public health advice to reduce the impact of health inequalities with sepsis.

## Methods

### Eligibility criteria

Peer-reviewed journal articles published between 01/01/2010 and 31/01/2023, written in English, were eligible for inclusion. Included studies had to be observational in design where the main outcome was either incidence or risk of sepsis (in the general population or hospital admissions) or sepsis-associated mortality. We included studies where the aim was assessing the impact of one of the following health inequality factors: socioeconomic, race/ethnicity, community, medical vulnerability, or pregnancy. Studies were excluded if they were conducted in a low- or middle-income country (LMIC) (according to the World Bank, to minimise differences in healthcare systems), were not observational in design (intervention studies or qualitative studies), full text was not available, or abstract was published in conference proceedings.

### Study selection

The database Embase (accessed through Ovid, last searched 25/03/3023) was used to search for relevant articles. Separate searches were carried out using the following terms in the titles of articles: sepsis OR septic in combination with one of the following groups of terms:

- Socioeconomic factors – depriv* or socioeconomic or socio-economic or socio or social or SES or IMD or income or occupation or education.
- Race/ethnicity factors – race or racial or ethnic* or minorit*.
- Community factors – urban* or rural or coast*.
- Medical vulnerability factors – residen* or care home or nursing home or care facility or living or social care or drug* or alcohol or disabil* or vulnerab*.
- Pregnancy – pregnan* or matern* or “post-partum” or “postpartum”.

**Fig. 1:**
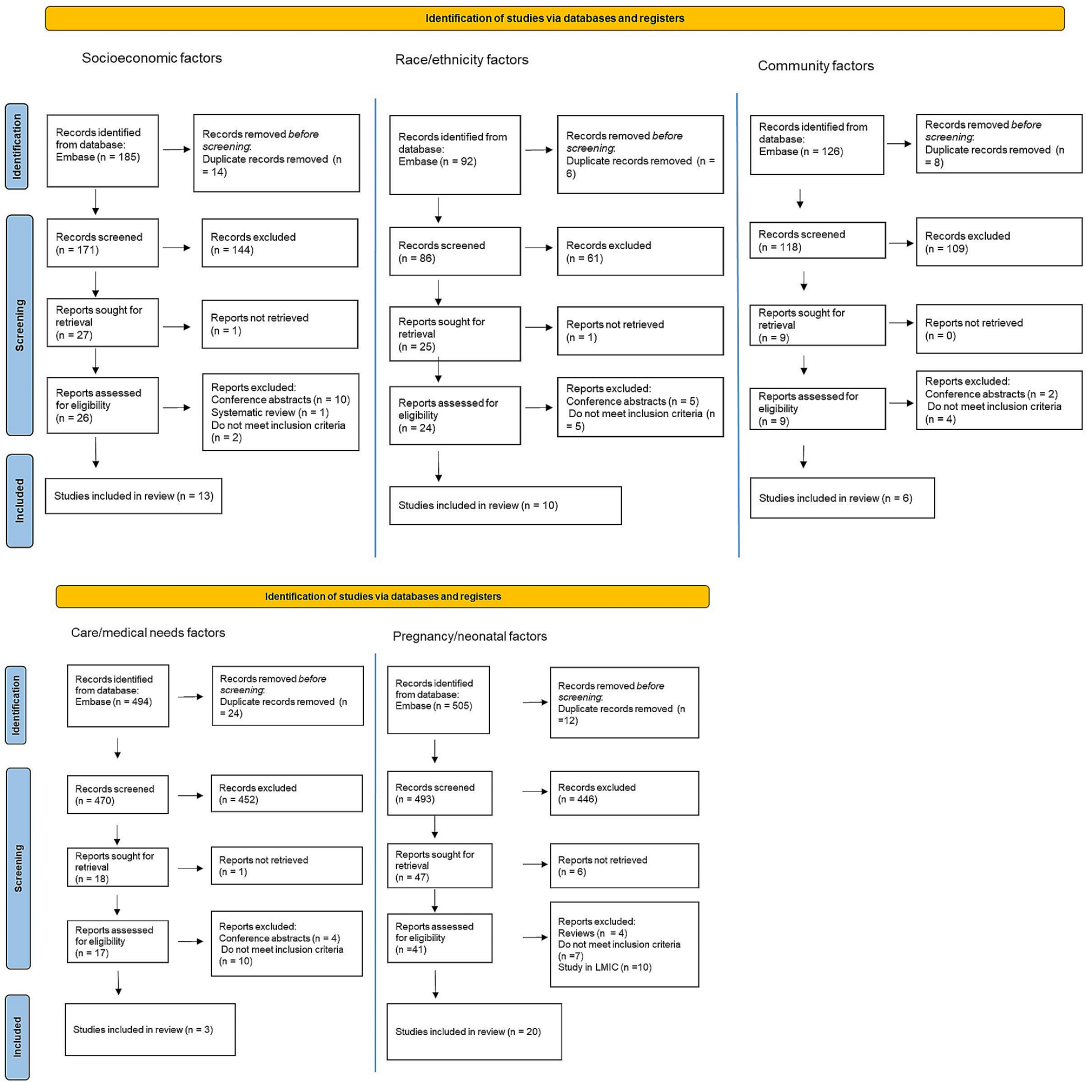
Flowchart showing search results and screening of studies. Five separate searches were conducted. After screening 53 papers were included

Duplicated articles were removed. All articles identified in the search went through a title and abstract screening to exclude ineligible articles. A full article review was then performed on the remaining papers, with any ineligible articles identified during the full paper review being excluded. In accordance with the PRISMA guidelines, the reasons for exclusion at this stage were recorded (see Fig. 1). Any further duplicates (studies that appeared in multiple searches) were also removed. The searches were performed in Ovid and the results were downloaded to Mendeley Reference Manager to apply the inclusion/exclusion criteria. Data extracted from the eligible articles were stored in Microsoft Excel. The following information was extracted from each included paper: title, authors, year published, study design, country where study was conducted, data source, sepsis identification method, number of patients in sepsis cohort, factors associated with inequality used in study and how they are measured, outcome(s) assessed in the study and key findings of associations between the factors and outcomes. For reporting we referred to the PRISMA guidelines [16] for systematic reviews, however, as this is a rapid review not all items are relevant. Further details of the search strategy can be found in the supplementary information.

## Results

### Selection of sources of evidence

The five searches returned a total of 1,402 results (185 socioeconomic, 92 race/ethnicity, 126 community, 494 medical/care needs, 505 pregnancy). After deleting duplicates, 1,338 papers were screened on title and abstract, with 1,254 excluded. 108 papers underwent full article screening, after which 53 were eligible. Of these, there were 13 articles assessing socioeconomic factors, 14 race or ethnicity, 3 assessing community, 3 care/medical needs, and 20 assessing pregnancy and post-partum factors. As the searches and selection were conducted separately there were 3 papers duplicated between the searches, resulting in 50 unique papers to include. Flowcharts showing the selection process for each search are shown in Fig. 1.

### Characteristics of sources of evidence

Table 1 displays the characteristics of studies included in the review. The majority of the included studies (31 out of 50) used data collected in the United States of America (USA) [17–47], with four in the UK [48–51], three in Israel [41, 52, 53], two in Canada [54, 55], one in Australia [56] and the others in Europe [57–63] or not specified [64, 65].Most of the studies used hospital or ICU discharge databases [17, 21, 23–25, 28–31, 36–39, 41–45, 47, 50, 60], other sources included national birth or obstetric registries [48, 49, 51, 62, 63], death registry datasets [27, 35], secondary analysis on data collected for other cohort studies [18, 26, 61], and a US cities public health dataset [22]. Six identified sepsis in neonatal patients or infants [21, 47, 53, 55, 56, 63] and another only included children aged between 0 and 20 years [28]. The rest either specified adults only or did not specify any age restrictions for the cohorts.

**Table 1:** Characteristics of all included studies in the review

Title	Authors	Year	Country	Data source	Population	Sepsis Identification	Factors associated with inequality	Outcome (s)	Sepsis cohort size
Association between sepsis incidence and regional socioeconomic deprivation and health care capacity in Germany - an ecological study	Rose N; Matthaus-Kramer C; Schwarzkopf D; Scherag A; Born S; Reinhart K, Fleischmann-Struzek C	2021	Germany	Inpatient database (DRG) covering all acute-care hospitals in Germany (except prison hospitals and psychiatric facilities)	Inpatient admissions in 2016, do not specify age restrictions	Explicit ICD-10 codes and Angus ICD-10 code criteria	Socioeconomic	Crude and age-standardised incidence of sepsis per district in 2016	146,985
Association of neighbourhood socioeconomic status with risk of infection and sepsis	Donnelly JP; Lakkr S; Judd SE; Levitan EB; Griffin R; Howard G; Safford MM; Wang HE	2018	USA	Reasons for Geographic and Racial Differences in Stroke (REGARDS) study	Hospital admissions between 2003–2012, adults aged over 45	Chart review using Sepsis-3 criteria	Socioeconomic	Hospital admissions and ED visits for serious infection/sepsis	964
Black-white racial disparities in sepsis: a prospective analysis of the Reasons for Geographic And Racial Differences in Stroke (REGARDS) cohort	Moore J; Donnelly J; Griffin R; Safford M; Howard G; Baddley J; Wang H	2015	USA	Reasons for Geographic and Racial Differences in Stroke (REGARDS) study	Hospitalisations for adults over 45 between 2003–2012	SIRS criteria	Race/ethnicity	Rates of sepsis	1,526
Socioeconomic status and risk of intensive care unit admission with sepsis	Storm, L; Schegelsberg, A; Andersen, LW; Jessen, MK; Kirkegaard, H	2018	Denmark	Tertiary ICU records	Tertiary ICU admissions with, 2008–2010, adults over 18 years old. Matched to up to 10 controls from background population on age, sex & zip code	severe sepsis or septic shock, not stated how identified	Socioeconomic	Admission to ICU with sepsis	383
Infection rate and acute organ dysfunction risk as explanations for racial differences in severe sepsis	Mayr F; Yende S; Linde-Zwirble W; Peck-Palmer O; Barnato A; Weissfeld L; Angus D	2010	USA	Hospital discharge databases of 7 US states	Admissions for all ages with severe sepsis in 2005	ICD-9 codes	Race/ethnicity	Incidence rates of severe sepsis hospitalisations.	381,787
Direct and indirect effects of socioeconomic status on sepsis risk and mortality: a mediation analysis of the HUNT study	Stensrud VH; Gustad LT; Damas JK; Solligard E; Krokstad S; Nilsen TIL	2023	Norway	Population-based HUNT studies	Adults aged between 20 and 70 admitted to hospital, 1995–1997 and 2006–2008	ICD-9 & ICD-10 codes, both explicit and implicit list	Socioeconomic	Sepsis and sepsis-attributable mortality	4,200
Where you live matters: the place of residence on severe sepsis incidence and mortality	Goodwin AJ; Nadig NR; McElligot JT; Simpson KN; Ford DW	2016	USA	Hospital discharge database in South Carolina	Adults (> 20 years of age) hospitalised with severe sepsis or septic shock	ICD-9 codes including Martin criteria	Care/medical	Age-adjusted severe sepsis incidence and in-hospital mortality rates	24,395
Socio-demographic characteristics associated with hospitalisation for sepsis among adults in Canada: a Census-linked cohort study	Hennessy DA; Soo A; Niven DJ; Jolley RJ; Posadas-Calleja J; Stelfox HT; Doig CJ	2020	Canada	National hospital discharge database linked to Canadian census	Adults over 18 years admitted with sepsis and severe sepsis, 2006–2009	ICD-10 codes	Socioeconomic	Hospital admission with a diagnosis of sepsis, secondary in-hospital death, admission to special care unit & LOS, hospital LOS, discharge disposition.	10,400
Racial differences in sepsis mortality at US academic medical center-affiliated hospitals	Chaudhary N; Donnelly J; Wang H	2018	USA	Hospital discharge data from Vizient, covers 120 medical centers and 300 hospitas in US.	Adults > 18 years old hospitalised with sepsis 2012–2014,	Angus ICD-9 codes	Race/ethnicity	Sepsis hospitalisation and hospital mortality.	1,114,386
Association of household income level and in-hospital mortality in patients with sepsis: a nationwide retrospective cohort analysis	Rush B; Wiskar K; Celi LA; Walley KR; Russell JA; McDermid RC; Boyd, JH	2018	USA	Nationwide Inpatient Sample (NIS), covers 20% of national hospital admissions	Identified patients over 18 years with sepsis admission in 2011	Angus ICD-10 code criteria	Socioeconomic	In-hospital mortality	671,858
Impact of socioeconmic status on mortality and unplanned readmission in septic intensive care unit patients	Schnegelsberg, A; Mackenhauer, J; Nibro, HL; Dreyer, P; Koch, K; Kirkegaard, H	2016	Denmark	Single ICU records	Tertiary ICU admissions with, 2008–2010, adults over 18 years old	severe sepsis or septic shock, not stated how identified	Socioeconomic	30-day mortality after ICU admission; 180-day mortality after hospital discharge, 180-day unplanned readmission after discharge	387
Lower socioeconomic factors are associated with higher mortality in patients with septic shock	Hidalgo DC; Tapaskar N; Rao S; Masic D; Su A; Portillo J; Rech M	2021	USA	Two hospitals dicharge records	Hospital admissions between 2017–2019, adults aged over 18	Sepsis-3 criteria for septic shock	Socioeconomic	30-day mortality, ICU length of stay, hospital length of stay	362
Race does not impact sepsis outcomes when considering socioeconomic factors in multilevel modeling	Vazquez Guillamet MC; Dodda S; Liu L; Kollef MH; Micek ST	2022	USA	Single centre records	Admissions with sepsis or septic shock, all adults (age not specified) 2010–2017	ICD-9 and ICD-10 codes	Socioeconomic; race/ethnicity	In-hospital mortality, hospital LOS, use of vasopressors, use of mechanical ventilation	11,432
Race, income and insurance status affect neonatal sepsis mortality and healthcare resource utilisation	Bohanon FJ;. Lopez ON; Adhikari D; Mehta HB; Rojas-Khalil Y; Bowen-Jallow, KA; Radhakrishnan, RS	2018	USA	Healthcare Cost and Utilisation Project’s (HCUP) Kids Inpatient Database (KID)	Sepsis admissions in neonates (< 28 days old) in 2006, 2009 & 2012.	ICD-9 codes	Socioeconomic; race/ethnicity	In-hospital mortality, LOS and total costs	116,882
Association between race and case fatality rate in hospitlisations for sepsis	Sandoval E; Chang DW	2016	USA	Healthcare Cost and Utilization Project (HCUP) State Inpatient Database (SID) and California OSHPD Patient Discharge Pivot Database	Adults over 18 years admitted in 2011	ICD-9 codes Martin criteria	Race/ethnicity	Case-fatality rate	131,831
Association of gender, age and race on renal outcomes and mortality in patients with severe sepsis and septic shock	Cerceo E; Rachoin JS; Gaughan J; Weisberg L	2021	USA	National Inpatient Sample (NIS)	Patients discharged between 2005–2014 after admission for severe sepsis or septic shock	ICD-9 codes for severe sepsis or septic shock	Race/ethnicity	In-hospital mortality	1,064,790
Ethnicity and sepsis characteristics and outcomes. Population based study	Karp G; Perl Y; Fuchs L; Almog Y; Klein M; Vodonos A; Drieher J: Talmor D; Codish S; Novack V	2013	Israel	Single ICU records, sepsis id’d with ICD-9 codes, between 2002–2008	All adults aged over 20 with sepsis admission, 2002–2008	ICD-9 codes	Race/ethnicity	In-hospital/28 day mortality	1,542
Impact of older age and nursing home residence on clinical outcomes of US emergency department visits for severe sepsis	Ginde A; Moss M; Shapiro N; Schwartz R	2013	USA	National Hospital Ambulatory Medical Care Survey (NHAMCS)	Adults over 18 years with sepsis ED visits, 2005–2009	Angus ICD-9 codes	Care/medical	In-hospital mortality, hospital LOS, admission to ICU	350,000 (estimate)
Hospital outcomes for children with severe sepsis in the USA by race or ethnicity and insurance status: a population-based, retrospective cohort study	Mitchell H; Reddy A; Montoya-Williams D; Harhay M; Fowler J; Yehya N	2020	USA	Healthcare Cost and Utilization Project (HCUP) Kids Inpatient Database (KID), covering 4200 US hospitals	Patients aged 0–20 years with severe sepsis in 2016	ICD-10 codes	Race/ethnicity	In-hospital mortality, hospital LOS (death as competing risk, censored at 30 days)	9,816
Racial disparities in sepsis-related in-hospital mortality: using a broad case capture method and multivariate controls for clinical and hospital variables, 2004–2013	Jones J; Fingar K; Miller M; Coffey R; Barrett M; Flottemesch T; Heslin K; Gray D; Moy E	2017	USA	Healthcare Cost and Utilization Project (HCUP) State Inpatient Database (SID)	Sepsis admissions, no age restrictions	Angus ICD-9 codes	Race/ethnicity	In-hospital mortality rates per 1,000 total sepsis hospitalisations	16,779,820
Treatment in disproportionately minority hospitals is associated with increased risk of mortality in sepsis: a national analysis	Rush B; Danziger J; Walley KR; Kumar A; Celi LA	2020	USA	National Inpatient Sample (NIS)	Sepsis admissions between 2008–2014, adults over 18 years	Angus ICD codes (doesn’t specify version)	Race/ethnicity	In-hospital mortality	4,221,221
Temporal trends in rural vs. urban sepsis-related mortality in the United States, 2010–2019	Oud L; Garza J	2022	USA	CDC and Prevention Wide Ranging Online Data for Epidemiological Research Multiple Cause of Death dataset	Sepsis-related deaths in US from 2010–2019	not stated	Community	Age-adjusted mortality rates per 100,000 population	
The effect of community socioeconomic status on sepsis-attributable mortality	Galiatsatos P; Brigham EP; Pietri J; Littleton K; Hwang S; Grant MC; Hansel NN; Chen ES	2018	USA	Neighborhood Health Profiles for Baltimore City, includes demographic & outcome data for 55 community statistical areas.	2017	not stated	Socioeconomic	Sepsis-attributable mortality	
Disparities in sepsis mortality by region, urbanisation, and race in the USA, a multiple cause of death analysis	Ogundipe F; Kodadhala V; Ogundipe T; Mehari A; Gillum R	2019	USA	CDC multiple cause of death data	Sepsis deaths in people aged 15 or over, between 2013 and 2016	ICD-10 codes	Race/ethnicity; community	Age-adjusted sepsis death rates	746,725
Rural patients with severe sepsis or septic shock who bypass rural; hospitals have increased mortality: an instrumental variables approach	Mohr N; Harland K; Shane D; Ahmed A; Fuller B; Ward M; Torner J	2017	USA	Administrative claims from emergency departments of midwestern state	All adults over 18 between 2005 and 2014	ICD-9 codes	Community	Hospital mortality, subsequent interhospital transfer, hospital LOS	13,461
Sepsis survivors admitted to nursing facilities: cognitive impairment, activities of daily living dependence, and survival	Ehlenbach W; Gilmore-Bykovski A; Repplinger M; Westergaard R; Jacobs E; Kind A; Smith M	2018	USA	Centers for Medicare and Medicaid Services (CMS) Chronic Conditions Data Warehouse (CCW)	People aged > 65 with severe sepsis episode, 2005–2009	Angus ICD-9 codes	Care/medical	1- year mortality, cognitive impairment, ADL	175,755
Race and sex based disparities in sepsis	Engoren M; Arslanian-Engoren C	2022	USA	Michigan hospital administrative dataset	First episode sepsis between 2009–2019, adults over 18 years	Sepsis-3 criteria	Race/ethnicity	90-day mortality, mechanical ventilation, RRT, time to initial antibiotic, post-sepsis hospital stay	34,999
Racial disparities in readmissions following initial hospitalisation for sepsis	Lizza B; Betthauser K; Juang P; Hampton N; Lyons P; Kollef M; Micek S	2021	USA	Tertiary care referral center	Patients hospitalised for severe sepsis and septic shock (and survived) between 2010–2017	ICD-9 and ICD-10 codes for severe sepsis and septic shock.	Race/ethnicity	All-cause readmission, sepsis readmission, postdischarge death.	3,390
The association between neighborhood socioeconomic disadvantage and readmissions for patients hospitalised with sepsis	Galiatsatos P; Follun A; Alghanim F; Sherry M; Sylvester C; Daniel Y; Chanmugam A; Townsend J; Saria S; Kind AJ; Chen E	2020	USA	Single hospital records	Sepsis admissions who survived to discharge, adults over 18 years in 2017	ICD-10 codes then reviewed charts for Sepsis-3 criteria	Socioeconomic	30-day readmission	531
Inclusion of social determinants of health improves sepsis readmission prediction models	Amrollahi F; Shashikumar S; Meier A; Ohno-Machado L; Nemati S; Wardi G	2022	USA	AllofUs study covering 35 hospitals	Patients over 18 years old admitted to hospital between 2017 and 2021	Sepsis-3 criteria	Socioeconomic	30-day unplanned readmission in sepsis patients	8,935
Risk factors, etiologies, and screening tools for sepsis in pregnant women: a multicenter case-control study	Bauer M; Housey M; Bauer S; Behrmann S; Chau A; Clancy C; Clark E; Einav S; Langen E; Leffert L; Lin S; Madapu M; Maile M; Mcquaid-Hanson E; Priessnitz K; Sela H; Shah A; Sobolewski P; Toledo P; Tsen L; Bateman B	2019	USA/Israel	Admissions data from 7 hospitals	Delivery admissions between 1994–2012 (depending on centre), matched 1:4 to non-sepsis controls	ICD-9 codes for sepsis, severe sepsis or septic shock, with manual chart review	Pregnancy	Risk factors	82
Severe sepsis in women with group B Streptococcus in pregnancy: An exploratory UK national case-control study	Kalin A; Acosta C; Kurinczuk J; Brocklehurst P; Knight M	2015	UK	UK Obstetric Surveillance System	All cases of severe maternal sepsis caused by Group B *Streptococcus* (GBS), 2011–2012, matched with controls who were non-sepsis deliveries	Prospective chart review, severe sepsis with laboratory confirmation of GBS	Pregnancy	Incidence of GBS sepsis, risk factors	30
The continuum of maternal sepsis severity: incidence and risk factors in a population-based cohort study	Acosta C; Knight M; Lee H; Kurinczuk J; Gould J; Lyndon A	2013	USA	California Vital Statistics records linked with statewide hospital discharge data	Maternal sepsis cases among all in-hospital live births, 2005–2007	ICD-9 codes	Pregnancy	Incidence of sepsis and severe sepsis, risk factors	1,598
Maternal obesity, obstetric interventions and post-partum anaemia increase the risk of post-partum sepsis: a population-based cohort study based on Swedish medical health registers	Axelsson D; Blomberg M	2017	Sweden	Medical Birth Registry, National Patient Register and Prescribed Drug Register	Maternal sepsis in women who gave birth between 1997–2012	ICD-10 codes	Pregnancy	Incidence and risk factors for post-partum sepsis	376
Severe maternal sepsis in the UK, 2011–2012: A National Case-Control Study	Acosta C; Kurinczuk J; Lucas D; Tuffnell D; Sellers S; Knight M	2014	UK	UK Obstetric Surveillance System	Maternal severe sepsis or septic shock, 2011–2012, matched to non-sepsis controls who delivered immediately before in same hospital	Prospective chart review	Pregnancy	Incidence and risk factors for severe maternal sepsis	365
Maternal sepsis incidence, aetiology and outcome for mother and fetus: a prospective study	Knowles S; O’Sullivan N; Meenan A; Hanniffy R; Robson M	2015	Ireland	Hospital records from two maternity hospitals	Pregnant and postpartum women with maternal sepsis between 2005–2012	Prospective chart review identifying primary or secondary BSI	Pregnancy	Incidence, maternal mortality and neonatal mortality	272
Case fatality and adverse outcomes are reduced in pregnant women with severe sepsis or septic shock compared with age-matched comorbid-matched nonpregnant women	Kidson K; Henderson W; Hutcheon J	2018	USA	National Inpatient Sample	Severe sepsis or septic shock in pregnancy admissions, women aged between 15–44 years, between 1998–2012. Compared with severe sepsis in non-pregnant women of the same age	ICD-9 codes	Pregnancy	Case fatality rates in pregnancy associated severe sepsis (PASS) and non-PASS (NPSS), hospital LOS	5,968
Maternal morbiditiy and mortality from severe sepsis: a national cohort study	Acosta C; Harrison D; Rowan K; Lucas D; Kurinczuk J; Knight M	2016	UK (excluding Scotland)	Intensive Care National Audit & Research Centre (ICNARC) Case Mix Programme	Severe sepsis in pregnant or recently pregnant women, admitted to critical care, between 2008–2010	Infection, at least 3 SIRS criteria and at least one organ dysfunction	Pregnancy	Maternal critical care admission rates, risk factors and death rates	646
Contemporary trends of reported sepsis among maternal decedents in Texas: a population-based study	Oud L	2015	USA	Texas Inpatient Public Use Data File (TIPUDF), covers all state-licensed hospitals	Pregnancy associated hospitalizations between 2001–2010	ICD-9 codes, for septic shock or infection (SIRS) in combination with organ failure	Pregnancy	Annual rate of sepsis among maternal decedents	131
Mortality associated with severe sepsis among age-similar women with and without pregnancy-associated hospitalization in Texas: a population-based study	Oud L	2016	USA	Texas Inpatient Public Use Data File (TIPUDF), covers all state-licensed hospitals	Pregnancy associated hospitalizations between 2001–2010 in women aged 20–34, compared with non pregnancy-associated hospitalizations	ICD-9 codes, for septic shock or infection (SIRS) in combination with organ failure	Pregnancy	Hospital mortality, number and type of failing organs	449
Progression from severe sepsis in pregnancy to death: a UK population-based case-control analysis	Mohamed-Ahmed O; Nair M; Acosta C; Kurinczuk J; Knight M	2015	UK	UK Obstetric Surveillance System and Confidential Enquiry into Maternal Death (CEMD)	Maternal deaths from non-influenza sepsis, compared with women who survived, between 2009–2012	Prospective chart review	Pregnancy	Risk factors associated with sepsis death	401
The sepsis in obstetrics score: a model to identify risk of morbidity from sepsis in pregnancy	Albright C; Ali T; Lopes V; Rouse D; Anderson B	2014		Discharge records from single centre	Pregnant and postpartum women presenting at ED with suspected SIRS or sepsis, between 2009 to 2011	SIRS with swab confirmation	Pregnancy	Morbidity - ICU admission within 48 h of presentation to ED, mortality, adverse perinatal outcome	850
Severe sepsis and septic shock in pregnancy: indications for delivery and maternal and perinatal outcomes	Snyder C; Barton J; Habli M; Sibai B	2013		Hospital records from two hospitals	Obstetric patients treated in ICU between 1995 to 2010 with severe sepsis or septic shock	ICD-9 codes confirmed with chart review	Pregnancy	Mortality, morbidity	30
Perinatal outcomes among patients with sepsis during pregnancy	Blauvelt C; Nguyen K; Cassidy A; Gaw S	2021	USA	Hospital records from single center	Patients who delivered at 20 weeks gestation or later, with antepartum clinical concern for sepsis discharged before delivery, 2012–2018	ICD-9 and ICD-10 codes	Pregnancy	Composite perinatal outcome of 1 or more of the following: fetal growth restriction, oligohydramnios, hypertensive disease of pregnancy, cesarean delivery, infant born small for gestational age, or stillbirth	59
Correlation of bacterial type and antibiotic sensitivity with antibiotic exposure in early-onset neonatal sepsis	Bromiker R; Ernest N; Meir M; Kaplan M; Hammerman C; Schimmel M; Schlesinger Y	2013	Israel	Hospital records from single center	Infants born betweem 1997–2007 with early onset neonatal sepsis	Culture confirmed infection	Pregnancy	Isolates with antibiotic resistance, Gram-negative isolates	94
Is peri-partum maternal fever alone a reliable predictor of neonatal sepsis? A single-centre, retrospective cohort study	Gupta S; Forbes-Coe A; Rudd D; Kandasamy Y	2021	Australia	Hospital records from single center	Term infants admitted to neonatal ICU with sepsis, between 2015 to 2020	Culture-proven neonatal bacteraemia and sepsis	Pregnancy	Predictors of EONS	14
Early-onset sepsis: a predictive model based on maternal risk factors	Puopolo K; Escobar G	2013	USA	Hospital records from multiple centers	Live births > = 34 weeks gestation with EONS from 1995–2007, matched to controls		Pregnancy	Predictors of EONS	350
Maternal obesity and risk of early onset neonatal bacterial sepsis: nationwide cohort and sibling-controlled studies	Villamor E; Norman M; Johansson S; Cnattingius S	2020	Sweden	Medical Birth Register	Live births > = 22 weeks, between 1997–2016 with EOS admission to neonatal unit within 72 h of birth	ICD-10 codes	Pregnancy	Incidence and risk factors of EONS	2,913
Maternal antibiotic exposure and risk of antibiotic resistance in neonatal early-onset sepsus: a case-cohort study	Wright A; Unger S; Coleman B; Lam P; McGeer A	2012	Canada	Hospital records from single center	Admissions to neonatal ICU within 24 h after birth, between 2008–2010	Confirmed serious bacterial infection	Pregnancy	Antibiotic resistance in EONS	60

ED – emergency department, LOS – length of stay, RRT – renal replacement therapy, EONS – early onset neonatal sepsis, ICU – intensive care unit, ADL – activities of daily living

The most common method of identifying sepsis in the studies was based on ICD codes. ICD-10 codes were used in twelve studies [17, 20, 27, 28, 32, 40, 46, 54, 60–63], ICD-9 codes were used in twenty-one studies [20, 21, 24, 25, 29, 31–33, 36–39, 41–46, 52, 61, 65] and one study did not specify which ICD version [34]. Of these studies, some used specific codes for sepsis, severe sepsis or septic shock whilst others used more comprehensive sets of codes including the Angus criteria [66] or Martin criteria [67]. Both the Angus and Martin methods include the sepsis specific codes and non-specific ICD codes for infection in combination with a code for organ dysfunction. Five studies [18, 19, 22, 23, 30] used the 2016 International Consensus definition for sepsis, otherwise known as the Sepsis-3 criteria [1]. Other studies used Systemic Inflammatory Response Syndrome with [50] and without criteria for organ dysfunction [26, 64], medical chart review [48, 49, 51, 53, 55, 56, 59] or did not specify [22, 35, 47, 57, 58]. The size of the sepsis patient cohorts varied from 14 [56] to 16,779,820 [[33] with a median cohort size of 2,913.

Regarding outcomes, 15 studies assessed the incidence or risk of sepsis [18, 26, 29, 31, 37, 41, 42, 48–50, 54, 57, 59–62], 23 studies looked at in-hospital (or short-term) mortality [17, 19–21, 24, 25, 28, 31, 33, 34, 36, 37, 39, 43–45, 50–52, 54, 58, 59, 65], four studies assessed mortality after hospital discharge [30, 32, 38, 58], five studies assessed hospital readmission rates after discharge [23, 30, 32, 40, 58] and four studies calculated population-level sepsis mortality rates [22, 27, 35, 61]. Seven of the studies relating to pregnancy assessed adverse perinatal outcomes and incidence of sepsis in neonates [46, 47, 53, 55, 56, 63, 64].

### Results of individual sources of evidence

#### Socioeconomic factors

The most common socioeconomic factors were income, level of education, employment status, unemployment rate and poverty rate. Others included were insurance status, occupation, cohabitation status and access to healthcare. There was variation in whether these were recorded at an individual level or matched to local data based on small area geographic identifiers (ZIP/postcode), and whether they were summarised into an overall score or included as individual covariates.

Five studies assessed the impact of socioeconomic factors on sepsis incidence or risk of developing sepsis. Factors found to be associated with increased risk of sepsis included low income [54, 60], low education level [57, 60, 61], lower socioeconomic status [18], marital/living status [54, 57] not being in work [54, 57], lower class of occupation and those who receive social benefits [61]. Three studies assessed 30-day or in-hospital mortality, which all found lower income was associated with increased risk of mortality when compared to the highest income groups. Another study reported decreased odds for highest household income quartile compared to the lowest quartile [20]. Hidalgo et al. [19] reported that unemployment and a neighbourhood poverty rate > 10% were all predictive of greater 30-day mortality. One study calculated population sepsis mortality rates per 10,000 persons and compared between income and poverty levels. Low-income neighbourhoods had a death rate of 3.65 (inter-quartile range (IQR) 2.78–4.40) versus high income neighbourhoods 2.80 (IQR 2.05–3.55) and high poverty neighbourhoods 4.20 (IQR 2.90–5.30) versus low poverty neighbourhoods 2.90 (IQR 2.00-3.60) [22]. For longer-term outcomes, two studies assessed the impact of socioeconomic factors on 30-day readmission after discharge. Lower income, lack of health insurance [23] and being more socioeconomically disadvantaged [23] were found to be associated with increased risk.

#### Race & ethnicity

Of the 14 studies assessing the impact of race or ethnicity on sepsis, 13 were based in the USA. Five of these studies only included race categorised as white or black/African-American [25,26,29,31,32], whilst other studies included categories for Hispanic [21, 24, 27, 28, 33, 34], Asian-American [24, 30], Asian/Pacific Islander (API) or Native American [21, 28, 33] and other/unknown. The study by Rush et al. [34] used a different approach by classifying hospitals as non-minority or minority, if the patient population of the hospital was more than twice the geographical census division mean.

The studies by Chaudhary et al. [31], Mayr et al. [29] and Moore et al. [26] compared rates of sepsis amongst either black or white populations only. Chaudhary et al. reported a higher sepsis rate for white patients compared to black patients with 109.4 cases (95% CI 109.2-109.6) per 1,000 hospitalisations versus 106.7 cases (95% CI 106.3-107.1). Moore et al. also reported a higher incidence of sepsis in white patients (9.10 per 1,000 person years) compared to black patients (6.93 per 1,000 person years). Contrary to these, Mayr et al. found a 67% higher severe sepsis hospitalisation rate in black patients (9.4 per 1,000 population) than white (5.6 per 1,000 population). All three studies covered hospital admissions in multiple US states, but they did differ in the age of patients included and severity of sepsis.

Eight studies considered the impact of race on in-hospital mortality in sepsis patients, with mixed results. Three studies reported higher mortality rates or increased risk of mortality in black or African-American patients than white patients [25, 28, 33] whilst Sandoval et al. [24] reported higher case fatality rates in white patients (15.1%) compared to black (14.0%), Hispanic (13.8%) or Asian patients (16.2%). One study [33] reported increased mortality rates in Hispanic patients compared to white patients, but two other studies did not find significant differences [21, 28]. Rush et al. reported unadjusted mortality rates at non-minority hospitals of 11.1%, compared with 12.3% (*p* < 0.001) at minority black hospitals and 12.7% (*p* < 0.001) at minority Hispanic hospitals. The only non-USA based study was based in Israel. Karp et al. [52] found that differences in risk of in-hospital mortality between Bedouin Arabs and the Jewish population could be explained by differences in age and Charlson comorbidity score.

For longer-term outcomes, one study [30] reported small differences in 90-day mortality rates between African American (18%), Asian-American (19%) and white (22%) patients. Lizza et al. [32] reported black patients had significantly higher rates of all-cause readmission (71.1% vs. 60.8%, *p* < 0.001) and sepsis readmission (19.8% vs. 14.0%, *p* < 0.001) than white patients. However, rates of post-discharge death were similar (white patients 36.5% vs. black patients 36.7%, *p* = 0.876). Ogundipe et al. [27] calculated age-adjusted sepsis death rates in non-Hispanic black, non-Hispanic white and Hispanic populations and reported lower death rates in Hispanic populations than non-Hispanic populations.

#### Community factors

The three papers included in the review that looked at community factors were conducted in the USA and used different ways of measuring urbanicity or rurality. Oud et al. [35] compared age-adjusted sepsis mortality rates between rural and urban communities from 2010 to 2019. The study reported in 2019 the overall rural rate was 57.9 deaths per 100,000, but in urban areas it was 48.3 deaths per 100,000 population. This was not a consistent pattern when adjusting for race. For example, in non-Hispanic blacks the urban mortality rates were higher than the rural rates. Ogundipe et al. [27] found the highest age-adjusted sepsis death rates were in non-metropolitan areas for both non-Hispanic black (micropolitan area 120.4 per 100,000 population, non-core area 109.4 per 100,000) and non-Hispanic white populations (micropolitan area 67.6 per 100,000, non-core area 66.4 per 100,000). Mohr et al. [36] assessed whether there were differences in patients in rural areas who attended their local emergency department or who bypassed their local hospital and travelled further to present to a hospital of top-decile inpatient sepsis volume. Sepsis patients who bypassed their local hospital had increased odds of mortality, with an OR of 1.26 (95% CI 1.03–1.53).

#### Medical needs

The three studies that considered factors relating to additional medical needs each used different measures. Goodwin et al. [37] identified patients living in medically underserved areas (MUA’s) based on the ratio of primary care physicians per 1,000 population, infant mortality rate, the proportion of the population with income below the poverty level and the proportion of the population over 65 years of age. The study reported higher incidence of sepsis (8.6 vs. 6.8 admissions per 1,000 people, *p* < 0.01) and mortality rates (15.5 versus 11.9 deaths per 10,000, *p* < 0.01) in MUA residents compared to non-MUA. Ginde et al. [39] included residence in a nursing home prior to an emergency department visit for sepsis, and reported increased risk of mortality for nursing home residents (OR 3.1, 95% CI 1.2–7.8). The study by Ehlenbach et al. [38] found that sepsis patients not discharged to a skilled nursing facility (SNF) had a mortality rate of 35.6%, while those discharged to a SNF but whom had not been resident in an SNF prior to sepsis had a mortality rate of 43.2% and patients who had been in a SNF before and after sepsis had a mortality rate of 52.8%.

#### Pregnancy/maternity

Studies assessing incidence of maternal sepsis reported rates of severe sepsis of 1.00 per 100,000 [48] maternities, 4.7 per 10,000 [49] maternities and 4.9 per 10,000 live births [42]. Estimates of non-severe sepsis included 198.69 per 100,000 [59] maternities, 2.4 per 10,000 women [62] and 10 per 10,000 live births [42]. Acosta et al. [50] estimated the absolute risk of maternal critical care unit admission with severe sepsis was 4.1 per 10,000 maternities (95% CI 2.9–5.6). Factors including increased BMI [41, 50, 62], older age [42, 50, 62], black and other ethnic minority race [49], increased levels of deprivation [50], African American race [41], pre-existing medical conditions [41, 49], complications of delivery and delivery via caesarean Sects [41, 49, 50, 59, 62] were found to be associated with an increased risk of developing maternal sepsis.

Two studies [43, 44] assessing mortality in maternity patients reported lower case-fatality rates in pregnancy associated severe sepsis (PASS) compared to non-pregnancy associated severe sepsis (NPSS). Maternal mortality rates in other studies varied, with reported rates of 10% [65], 10.7% [51], 1.8/100,000 maternities [50] and no deaths in one study [59]. Increased BMI [50], being in the most deprived two IMD quintiles [50], pre-existing medical conditions [51] and being multi-parous [51] was found to be associated with increased maternal mortality. Antepartum sepsis was found to be associated with increased risk of placental dysfunction and maternal ICU admission during delivery hospitalization [46]. Five studies considered outcomes relating to early onset neonatal sepsis (EONS), with reported rates of 1.03 cases per 1,000 live births [53] and 1.48 per 1,000 live births [63]. Risk factors associated with EONS were maternal exposure to antibiotics [47, 53, 55], maternal BMI [63], caesarean section delivery [63] and gestational age [47].

Supplementary Tables 1 and 2 show the findings from all included studies and can be found in additional file 1.

## Discussion

### Summary of evidence

Socioeconomic factors associated with increased incidence of sepsis included lower socioeconomic status, unemployment, and lower education level, although findings were not consistent across studies. Studies assessing the association between ethnicity and sepsis rates reported mixed results, with two studies finding increased sepsis rates in white populations compared to black populations and another showing higher rates in black populations than white. Living in a medically underserved area or being resident in a nursing home was also shown to increase risk of sepsis. In terms of mortality, lower income, unemployment, and poverty levels were all associated with increased in-hospital mortality. In studies considering effects of ethnicity on in-hospital and longer-term mortality the results were mixed, with some studies finding no significant associations, some reporting increased odds of mortality in non-white populations and others reporting increased mortality in white populations. Sepsis mortality rates were also found to be higher in people living in rural areas and those who were resident in a skilled nursing facility.

It is notable that the literature is dominated by research conducted in the USA and none of the studies identified under the non-pregnancy related searches used UK data. This is an important consideration for healthcare and public health professionals outside of the USA as differences in structural inequalities between the USA and other high-income countries may make the results less generalisable. The majority of studies focused on in-hospital mortality as the primary outcome, so there also needs to be more focus on the risks of developing sepsis and longer-term outcomes such as healthcare utilisation.

The sources of data varied between the studies, as did the methods of identifying sepsis. Differences in sepsis definitions leads to different reported prevalence/cohort sizes [68]. Some of the studies were based in single centres and only included a few hundred patients, whilst others represented national populations and included millions of patients. Many of the studies used data from secondary care only and none used primary care data, even though the majority of cases of sepsis develop in the community rather than the hospital. Additionally, there was a lot of variation in measures used in the analyses, particularly in the studies assessing socioeconomic factors, where there was no standardised definition of socioeconomic status and therefore results varied. There were some studies who assessed a combination of socioeconomic, community and race factors, however, some only focused on one area related to health inequality. This is important as there is overlap between the different areas. The paper by Vazquez Guillamet et al. [20] concluded that race did not have a significant effect on sepsis mortality when accounting for socioeconomic variables. A commentary piece published in 2018 by Shankar-Hari and Rubenfeld [69] titled “Race, ethnicity and sepsis: beyond adjusted odds ratios” suggested that there needs to be more research into the underlying causes of race/ethnicity disparities not just in sepsis but in wider health areas. Future studies should take into account not just socioeconomic status and population demographics, which will likely vary between ethnic groups, but also consider the intersectionality between these and other factors such as comorbidity levels and health behaviours e.g. smoking, alcohol use or exercise.

### Limitations

Due to the rapid nature of the review the scope was limited and the search strategy not as comprehensive as for systematic reviews. We searched for the key terms in the titles only, searched a single database (Embase) and only included studies published from 2010 onwards. We also acknowledge that pathogen specific publications which do not specifically include the word sepsis may have been screened out. Examples include those that report on invasive group A and B streptococcal disease [67]. Studies conducted in LMICs were excluded as the results will be less generalisable to the UK population. Whilst the burden of sepsis is highest in LMICs there is a lack of good quality data from these countries [70]. The challenges in recognising and managing sepsis within LMICs, such as lack of access to healthcare, malnutrition and infrastructure [71], are not as applicable in higher- income countries. Some aspects of the Core20PLUS5 approach to addressing health inequalities were not included in this rapid review. These mainly related to inclusion health groups, including people with multi-morbidities, vulnerable migrants, Gypsy, Roma and Traveller communities, sex workers, people in contact with the justice system and victims of modern slavery. Additionally, the 4 comorbidities/conditions within the ‘5’ component other than maternity were not areas of focus (severe mental illness, COPD, cancer & hypertension). The five included areas were chosen as they are the factors that cover the largest groups in the population and were identified as the most important. Although we did not include the other areas in our review it is still vital that future studies consider these aspects in order to address all potential influences on sepsis risks and outcomes. The bias of the included studies was not assessed, nor did we critically appraise them.

### Future work

From the studies identified, there are clear correlations between sepsis morbidity and mortality and the presence of factors associated with health inequalities. There is a need for UK based studies, using nationally representative data, to better understand how factors associated with health inequalities affect sepsis incidence and mortality in the UK population. With the availability of electronic health record data for research there are increasing opportunities to disaggregate the data and stratify risk by patient demographic. For example, in the UK the Clinical Practice Research Datalink (CPRD) and Hospital Episode Statistics (HES) data provide nationally representative primary and secondary care records with linkage available to deprivation and socioeconomic scores. Once this is better understood, healthcare and public health professionals can be empowered to close the health gap and reduce inequalities through targeted recommendations for the recognition and early management of sepsis. Recent guidance in the UK highlighted the importance of early intervention in sepsis whilst balancing that with the need to use antibiotics more appropriately. Understanding which patients are at greater risk of sepsis mortality and morbidity, in terms of the factors associated with inequalities discussed in this review and other known risk factors, may help clinicians target antibiotic use more effectively. Given the lack of evidence from outside the USA, there is not sufficient information available to inform policy, at either a global level or an individual country level (except the USA). Although some of the findings from USA studies may be generalisable to other settings there needs to be further exploration of the similarities and differences in inequality factors in different populations. Critical to the above is improved coding in electronic health records alongside appropriate data linkage.

## Conclusion

Factors relating to health inequalities such as deprivation and ethnicity have been shown to be associated with poorer outcomes in COVID-19 and increased rates of antimicrobial resistance. In order to inform local guidance and drive public health measures, there is a need for studies conducted across more diverse setting and countries.

### Electronic supplementary material

Below is the link to the electronic supplementary material.

**Additional file 1: Table 1**. Findings of studies assessing the impact of factors of health inequality on sepsis incidence or risk. **Table 2**. Findings of studies assessing the impact of factors of health inequality on sepsis mortality and other outcomes.

**Additional file 2**. Search strategy including PICO criteria and exact search terms.

## Data Availability

All data generated or analysed during this study are included in this published article and supplementary materials.
