# Supplementary material for: Rapid systematic review on risks and outcomes of sepsis: the influence of risk factors associated with health inequalities
Source: Int J Equity Health. 2024 Feb 21;23:34. doi: 10.1186/s12939-024-02114-6 (PMC10882893; doi:10.1186/s12939-024-02114-6)
Supplement: Supplementary file 2 — Additional file 2. Search strategy including PICO criteria and exact search terms. [file 12939_2024_2114_MOESM2_ESM.docx]

**Search Strategy**

**PICO criteria:**

Participants/population – human population of any age diagnosed with sepsis

Intervention(s), exposure(s) - factors associated with health inequality including socioeconomic factors, race/ethnicity, community, medical vulnerability and pregnancy/maternity

Comparator(s)/control - not applicable

Main outcome(s) – risk/incidence of sepsis or sepsis mortality

Additional outcomes - sepsis morbidity (e.g. long-term health impacts, quality of life)

**Inclusion/exclusion criteria:**

Studies will be screened according to the following inclusion & exclusion criteria:

Inclusion criteria -

Peer-reviewed journal articles published between 01/01/2010 and 31/01/2023

Observational studies

Conducted in a high-income country (according to the World Bank, https://data.worldbank.org/income-level/high-income)

Written in the English language

Meets PICO criteria stated above

Exclusion criteria –

Qualitative studies, systematic reviews, meta-analyses, RCTs or non-randomised intervention studies

Conference abstracts

Conducted in low- or middle- income countries (according to the World Bank, <https://data.worldbank.org/income-level/low-and-middle-income>)

Does not meet PICO criteria

**Searches:**

All five searches will be conducted in the Embase database, accessed through Ovid.

The search strings are as follows, all to be limited between 01/01/2010 and 31/01/2023

Search 1 – socioeconomic factors

1. (sepsis OR septic).m_titl
2. (depriv* OR or socioeconomic or “socio-economic” or socio or social or SES or IMD or income or occupation or education).m_titl
3. 1 and 2

Search 2 – race/ethnicity factors

1. (sepsis OR septic).m_titl.
2. (race OR racial OR ethnic* OR minorit*).m_titl.
3. 1 and 2

Search 3 – community factors

1. (sepsis OR septic).m_titl
2. (urban* OR rural OR coast*).m_titl
3. 1 and 2

Search 4 – medical vulnerability factors

1. (sepsis OR septic).m_titl
2. (residen* OR “care home” OR “nursing home” OR “care facility” OR “living” OR “social care OR drug* OR alcohol OR disabil* OR vulnerab*).m_titl
3. 1 and 2

Search 5 – pregnancy/maternity factors

1. (sepsis OR septic).m_titl
2. (pregnan* OR matern* or “post-partum” OR postpartum).m_titl
3. 1 and 2

All searches were last conducted on 25/03/2023.

**Data extraction:**

Study info – title, authors, year, country study carried out in

Data source/population – source of data used in study (e.g. hospital records, mortality records), population of interest (e.g. age range, inpatient or population-based cohort), sepsis identification method (e.g. ICD codes or Sepsis-3 criteria), cohort size

Inequality measure – list all relevant factors associated with health inequalities and how they have been measured/defined in the study

Outcome – list all outcomes and how they have been measured/defined

Key findings/results
